# How publish-or-perish culture drives evidence fragmentation in oncology

**DOI:** 10.1080/07853890.2026.2726583

**Published:** 2026-09-10

**Authors:** Minh Toan Chau, Bismark Bright Ofori-Manteaw, Jacob Leonard Ago, Kelly Bentley-Spuur, Haydn Kerr, Samuel White

**Affiliations:** ^a^ School of Dentistry and Medical Sciences, Charles Sturt University, Wagga Wagga, Australia; ^b^ Teaching Innovation in Radiography Group, Charles Sturt University, Wagga Wagga, Australia; ^c^ College of Health Sciences, Vin University, Vietnam; ^d^ Discipline of Medical Radiations, School of Health and Biomedical Sciences, RMIT University, Bundoora Campus, Australia; ^e^ Department of Medical Laboratory Technology, Faculty of Applied Sciences, Accra Technical University, Accra, Ghana; ^f^ South Australia Medical Imaging, Royal Adelaide Hospital, Adelaide, Australia; ^g^ Adelaide Medical School, Faculty of Health and Medical Sciences, University of Adelaide, Adelaide, Australia

Oncology sits at the centre of modern medicine and international healthcare systems. In 2022 alone, the world saw close to 20 million new cancer cases and 9.7 million cancer deaths [1]. Clinicians, guideline panels, and patients make high-stakes decisions in a rapidly evolving evidence ecosystem. Increasing use of publication and citation metrics within the research community may lead to inappropriate prioritization of research output volume over genuine impact [2–4]. This culture can incentivize publication of fragments of the same evidence base that when evaluated on an individual basis may resemble independent advances but when evaluated in totality reflect considerable duplication that offers minimal incremental benefit to evidence end users and patients. The concern is not secondary analysis itself, as complex oncology research programmes may legitimately generate several distinct outputs. Rather, evidence fragmentation arises when related publications are insufficiently linked, shared datasets or populations are not transparently disclosed, overlapping data are repeatedly analyzed without clear justification, or individual outputs offer minimal incremental value. This editorial examines how inappropriate evidence fragmentation occurs in oncology, how it differs from legitimate secondary publication, and how researchers, editors and institutions can improve transparency across related research outputs.

## Defining evidence fragmentation

In this paper, evidence fragmentation refers to the disaggregation of a single coherent research programme into multiple ‘minimal publishable units’, often without sufficient justification or transparency regarding shared datasets, hypotheses, or analytic overlap. Fragmentation exists on a spectrum. At one end, teams may publish justified post-hoc analyses that answer pre-specified questions with appropriate methodological and statistical considerations. At the other end, redundant publication and ‘salami slicing’, which describes the act of inappropriately splitting data derived from a single research idea into multiple smaller ‘publishable’ units or ‘slices’ [5]. This creates inflated research outputs while adding limited new knowledge or benefit [6]. These practices are further reinforced by publication bias, with the tendency for statistically significant findings to be preferentially published. This incentivizes the practice of data dredging, whereby datasets are repeatedly analyzed under multiple hypotheses until significant results emerge [7,8]. Such dynamics contribute directly to evidence fragmentation.

Researchers may choose to curate complex oncology datasets into smaller entities that can sustain individual publications [9]. This approach is not inherently unethical and may be required to address legitimately distinct scientific questions. However, when fragmentation is driven primarily by publication incentives rather than conceptual clarity, it can obscure the overall structure of the research programme and create ambiguity around published findings. We thus introduce the concept of the ‘minimal publishable unit’ to describe the outputs derived from inappropriate research fragmentation [10]. Importantly, fragmented publishing in oncology is not simply a matter of academic style. It has direct implications for how evidence is interpreted and applied. When related results are dispersed across multiple papers without clear linkage, readers may struggle to determine whether findings are derived from independent cohorts or from repeated analyses of the same population. This lack of transparency becomes particularly problematic in fields such as oncology, where evidence synthesis plays a central role in shaping clinical guidelines, treatment planning and decisions.

Oncology research is particularly vulnerable to fragmentation because of the scale and structure of its datasets. Large clinical trials, cancer registries, genomic consortia, and imaging repositories routinely generate multiple secondary analyses from the same cohort. These analyses often examine different endpoints such as survival, toxicity, biomarker associations, imaging features, or subgroup effects where each analysis may be valid in isolation. The literature suggests that if the ‘slice’ of a larger study tests a different hypothesis, addresses a substantially different question, has a different methodology or populations in contrast to the larger study, then it is acceptable to publish it separately [11]. However, it is important for authors to carefully consider each of their distinct research questions prior to data curation or ‘slicing’ to ensure the dataset is sufficiently representative of the clinical populations being assessed across the different papers. This can be achieved through synthesis of detailed study protocols. The number of publications arising from a study does not, by itself, determine whether fragmentation has occurred. The central questions are whether each report addresses a distinct scientific question, contributes meaningful new knowledge and transparently identifies its relationship to the parent study and related publications.

On the contrary, the cumulative effect of inappropriate salami slicing can be the dispersion of a single study across multiple publications with limited transparency regarding their shared origin. While the ethical dimensions of such practices have been widely discussed [12], the more immediate concern in oncology is their impact on the integrity of the evidence base and downstream implications on clinical decision-making

## When multiple publications are scientifically justified

Multiple publications arising from the same study, cohort or research programme are not inherently inappropriate. Complex oncology studies may produce distinct clinical, biological, imaging and patient-reported findings that cannot be communicated clearly within a single article. Separate publication may therefore improve interpretation, provided that each report has a distinct scientific purpose and remains transparently connected to the parent study [6,11,12]. Reporting outcomes at one, three and five years would not ordinarily constitute inappropriate fragmentation when each follow-up period provides clinically meaningful new information. Early reports may describe treatment response or acute toxicity, while later reports may examine survival, recurrence, late toxicity or durability of treatment benefit. Concern arises when similar findings are repeatedly presented as new studies, follow-up periods are selected primarily to generate publications, or earlier reports are not clearly identified [10,12,13].

The later reanalysis of existing biological samples using a new method, such as next-generation sequencing, would not automatically constitute duplicate publication. A separate report may be justified when the new technology generates materially different data, supports a distinct hypothesis or permits an analysis that was not previously possible. Authors should disclose the origin of the samples, cite previous analyses and explain the incremental contribution of the new method [14]. Trials may also contain several prespecified objectives that require separate publications. For example, treatment efficacy, quality of life and molecular correlates may each warrant a focused report. Each publication should include the trial registration number, identify the relevant protocol objective and cite other reports arising from the trial [15,16]. Publishing related objectives in different journals without such linkage can make the findings appear to represent independent studies. The opposite problem should also be recognized. Combining every clinical outcome, subgroup analysis, biomarker result and patient-reported measure into one publication may obscure the primary findings. The goal should therefore not be one study, one paper. A more appropriate principle is one coherent scientific question per transparently linked report. Separate publication is most defensible when it involves a distinct research question, a meaningful new outcome or method, limited repetition, clear identification of the parent study and transparent citation of related publications [6,11]. The Rectal Cancer and Pre-operative Induction Therapy Followed by Dedicated Operation (RAPIDO), trial provides an example of how a single oncology trial can generate a legitimate family of linked publications addressing distinct clinical, safety, imaging, pathological and patient-centred questions (Table 1).

**Table 1.** The RAPIDO trial as an example of a legitimate family of linked oncology publications.

Publication domain	Distinct purpose of the analysis	Example output from the RAPIDO trial	Features supporting separate publication
Primary clinical outcomes [17]	Evaluate the effectiveness of total neoadjuvant treatment compared with standard treatment	Disease-related treatment failure, distant metastasis, pathological complete response and adverse events	Primary trial report addressing the principal treatment comparison
Safety and treatment delivery [18]	Examine whether the treatment could be delivered safely and as planned	Treatment compliance, acute toxicity, surgical procedures and postoperative complications	Focused analysis of feasibility and treatment delivery rather than clinical effectiveness
Imaging quality [19]	Assess whether magnetic resonance imaging performed across participating centres met protocol requirements	Audit of required MRI sequences, slice thickness and technical compliance	Distinct imaging methodology and quality-assurance question
Pathological and correlative outcomes [20]	Examine pathological response and its association with subsequent outcomes	Characteristics associated with pathological complete response and oncological outcomes among complete responders	Focused analysis of a clinically meaningful subgroup and pathological endpoint
Patient-centred outcomes [21]	Assess treatment effects from the patient perspective	Health-related quality of life, bowel function and late toxicity	Use of patient-reported outcomes not fully represented by the primary clinical endpoints
Long-term follow-up [22]	Evaluate whether treatment effects and risks changed over time	Five-year risk and patterns of locoregional failure	Later follow-up provides clinically meaningful information unavailable in the initial report

All publications clearly identified the RAPIDO parent trial and addressed distinct questions using different outcomes or analytical approaches. Their shared origin does not make them fragmented publications. Rather, they represent legitimate secondary outputs when the common trial population is disclosed, related publications are cited and the incremental contribution of each analysis is clear.

## Fragmentation in clinical trials and observational research

### Clinical trials and selective outcome reporting

Inappropriate research fragmentation has been documented across biomedical research [9,23–25]. Analyses of randomized trials show that a single study can generate multiple publications without clear cross-referencing, making it difficult to distinguish independent evidence from repeated analyses of the same dataset [10,15,16,26]. In oncology, where trials frequently include extensive correlative science components, this issue is further amplified [16]. A single trial may yield separate papers on clinical outcomes, molecular profiling, imaging biomarkers, and exploratory subgroup analyses, each presented as a standalone contribution. When these outputs are not transparently linked, evidence synthesis becomes vulnerable to duplication, misclassification, and overestimation of effect sizes and precision [16].

Clinical trials illustrate how quickly one dataset can fragment. Across 191 randomized trials published in high-impact journals, 46% generated secondary or post-hoc publications, producing 475 additional papers within five years, with some trials yielding more than 10 and up to 51 secondary publications [10]. In oncology, selective and inconsistent reporting persists even when protocols are available. Studies comparing trial protocols, registry entries, and publications have demonstrated discrepancies in primary endpoints and incomplete reporting of secondary outcomes. These unplanned analyses are more likely to highlight positive findings [16,27]. When trials fragment in this way, meta-analysts and guideline panels may struggle to align the unit of evidence with the unit of analysis, increasing the risk of biased synthesis and misinformed clinical recommendations.

The consequences become particularly evident in the reporting of secondary endpoints. Secondary endpoints are additional outcomes measured in a study beyond the primary endpoint, often providing important information on treatment safety, tolerability, and patient-centred outcomes such as quality of life [28–30]. In an analysis of 280 phase III oncology trials, 2562 secondary endpoints were identified, yet only 69% were ever published, and consistency between protocols and trial registries was limited. Patient-reported outcomes and translational correlatives such as biomarker or mechanistic analyses linked to treatment response were among the least frequently reported. Best practice recommends that all pre-specified primary and secondary endpoints be clearly defined in study protocols and transparently reported, with any deviations explicitly justified. The under-reporting of patient-reported outcomes is especially concerning, given their importance in capturing treatment tolerability and quality of life. Contemporary methodological guidance such as that issued by GRADE advocates for patient-important outcomes to be included in evidence syntheses [31]. However, evidence suggests that such outcomes are inconsistently reported and remain underutilized in diagnostic and oncology research, limiting their integration into evidence synthesis and clinical practice guideline development [32,33].

### Observational, genomic and imaging research

Observational oncology research introduces an additional pathway for fragmentation. Cancer registries and large datasets enable rapid analyses but also increase the risk of overlapping populations being reported across multiple studies. The same cohort may appear in different publications with minor variations in eligibility criteria, timeframes, or endpoints. If such overlap is not recognized, meta-analyses may inadvertently double-count populations, inflating precision and distorting their estimates of effect. Empirical work in oncology and broader health research has demonstrated that violation of the statistical independence assumption underlying meta-analysis can reduce generalizability and increase false positive findings [34]. This again highlights the need for greater transparency and methodological vigilance when synthesizing evidence derived from large, shared datasets [35].

The expansion of biomarker and precision oncology research further intensifies this fragmentation. High-throughput genomic and proteomic datasets enable repeated hypothesis testing across the same cohort, contributing to a proliferation of small, incremental studies that often lack external validation [27,36,37]. Similar patterns are observed in medical imaging research, particularly in radiomics and artificial intelligence, where multiple predictive models are developed using overlapping datasets with only minor methodological variation [34,35,38]. Cancer prognostic marker studies show a long-standing skew toward statistically significant findings, which weakens the value of p-values for deciding which markers truly matter [39]. In breast cancer gene expression research, investigators demonstrated that random gene sets often appear associated with outcome, and many published signatures performed no better than expected by chance [40]. Radiomic features are similarly sensitive to image acquisition, reconstruction, preprocessing and software choices, with limited methodological consensus and variable reporting quality [41]. Audits of radiomics research have also highlighted recurring reporting and design limitations [42,43]. These practices can create the appearance of independent evidence while repeatedly drawing from the same underlying data, making evidence synthesis more difficult and slowing clinical translation. Reuse of data is not itself the problem. Concern arises when shared participants, samples or datasets are not disclosed and related analyses are presented as independent evidence.

Related research integrity threats may further complicate the oncology evidence base, although they should not be conflated with evidence fragmentation. Paper mills involve the fabrication, manipulation or commercial production of research outputs and authorship positions [44,45]. Generative AI presents a different concern. It may support legitimate activities such as language editing, summarization and manuscript preparation, but it may also increase the speed and scale of low-quality manuscript production when used without appropriate human oversight, verification and disclosure. These practices do not necessarily constitute evidence fragmentation, but they may increase the volume of unreliable or poorly connected evidence entering the literature and compound the challenges faced by evidence end users. Current ICMJE guidance requires authors to disclose the use of AI-assisted technologies and confirms that AI tools cannot meet authorship criteria [46].

## Consequences for evidence synthesis and clinical practice

The harm is practical with real world implications Duplicate and fragmented publications can bias meta-analyses, which can then impact guideline development and, ultimately, patient treatment and care. A landmark case study showed that covert duplicate publication led to a 23% overestimation of an intervention’s apparent benefit in meta-analysis [47]. Evidence synthesis standards address this risk directly. Preferred Reporting Items for Systematic reviews and Meta-Analyses (PRISMA) 2020 strengthens expectations for transparent reporting [48] indirectly supporting identification of overlapping studies, and Cochrane [49] requires reviewers to collate multiple reports so that each study, not each report, is the unit of interest [50]. Journal policies reinforce the same principle by warning that overlapping publications can distort evidence through double counting [46]. More specific reporting guidance may be required to support the identification and management of overlapping publications.

Readers, reviewers and meta-analysts can use several signals to identify publications arising from shared or overlapping datasets. These include identical or related trial registration numbers, overlapping recruitment periods, similar sample sizes, matching eligibility criteria, common institutional cohorts, repeated descriptions of participant characteristics, shared treatment protocols and substantial overlap in authorship. Titles and abstracts may not always disclose these relationships clearly, so protocols, trial registries, supplementary materials and cited companion publications may require review. No single feature confirms dataset overlap. These signals should be considered collectively before reports are treated as independent studies in evidence synthesis. Reducing inappropriate evidence fragmentation requires coordinated action across the research lifecycle. Table 2 summarizes practical responsibilities for researchers, reviewers, editors, evidence synthesis teams, institutions and funders.

**Table 2.** Stakeholder actions to reduce inappropriate evidence fragmentation.

Stakeholder	Recommended actions	Intended outcome
Researchers and authors	Prespecify distinct research questions and analyses; disclose shared cohorts, datasets and biological samples; identify the parent study; provide trial registration numbers; cite related publications; explain the incremental contribution of each report	Improve transparency and help readers distinguish legitimate secondary analyses from redundant publication
Peer reviewers	Assess whether the research question is sufficiently distinct; compare the submission with cited and related outputs; examine similarities in cohorts, recruitment periods, sample sizes, eligibility criteria, outcomes and methods; request clarification where overlap is unclear	Identify unnecessary duplication and ensure that secondary analyses provide meaningful new knowledge
Journal editors	Require disclosure of related manuscripts and publications; verify trial and protocol identifiers; request statements describing dataset overlap; apply clear policies on secondary analyses, overlapping publications and salami slicing	Strengthen editorial oversight and improve linkage between related reports
Systematic reviewers and meta-analysts	Search for companion publications; compare trial identifiers, recruitment periods, institutions, authorship and participant characteristics; group multiple reports under the parent study; avoid counting the same participants more than once	Preserve statistical independence and reduce bias from duplicated or overlapping populations
Research institutions	Assess research quality, transparency, collaboration, data sharing and reproducibility rather than publication counts alone; provide training in responsible publication planning	Reduce incentives for minimal publishable units and support coherent research programmes
Funders	Require publication and dissemination plans; support protocol registration, data stewardship and long-term follow-up; monitor whether funded datasets generate transparent and reusable outputs	Promote complete reporting and maximize the value of funded research
Publishers and journals	Enable linking of related articles, protocols, datasets and corrections through persistent identifiers; require accessible supplementary material and data-sharing statements	Make publication families easier to identify and navigate

## Improving linkage, transparency and research assessment

Solutions exist, but oncology requires a coordinated approach across the research lifecycle. At the study design stage, researchers should plan analyses *a priori*, defining distinct research questions before dataset assembly. This facilitates appropriate study design, ensures adequate statistical power for each analysis, and reduces the likelihood of inappropriate post hoc fragmentation driven by publication incentives. At the reporting level, traceability is essential. The International Committee of Medical Journal Editors (ICMJE), requires trial registration at or before enrolment [51], while World Health Organization (WHO) guidance frames trial registration and results disclosure as ethical and scientific responsibilities, including the use of trial identifiers to enable linkage across publications [52–54]. Editors can strengthen this linkage by requiring registration identifiers in abstracts, mandating protocol and statistical analysis plan availability, and requiring authors to disclose prior publications, secondary analyses, and shared datasets. The Consolidated Standards of Reporting Trials (CONSORT) remain the baseline for transparent reporting [55].

Improving evidence linkage also depends on data transparency and collaboration. The ICMJE requires data sharing statements for clinical trial reports [56], yet oncology data and code sharing remain limited, and datasets meeting core FAIR (Findable, Accessible, Interoperable, Reusable) principles are rare [57]. Expanding cross-institutional and international collaboration, alongside the development of living evidence approaches that continuously update and integrate findings, may help reduce duplication and promote more coherent evidence generation.

Journals can further move from policy to enforcement by verifying that datasets, code, and protocols are not only declared but accessible and usable. Persistent identifiers can support linkage across outputs. The Open Researcher and Contributor ID (ORCID) provides a unique identifier for researchers [58], while the Contributor Role Taxonomy (CRediT) enhances transparency in authorship contributions within large research teams [59].

Oncology will not fix evidence fragmentation without changing how fragmentation may benefit or reward researchers. Declaration on Research Assessment (DORA) and the Leiden Manifesto reject journal metrics as a shortcut for quality and call for assessment that values content, transparency, and responsible practice [2,3]. The Coalition on Advancing Research Assessment (CoARA) extends this direction with commitments to reform assessment toward quality and broader contributions [4]. If institutions count metrics such as papers and citations, researchers will keep ‘slicing’. If institutions reward integrated trial reports, careful synthesis, shared data, and reproducible analysis, the literature can grow in value without inflating in noise.

The credibility and clinical value of oncology research depend on an evidence base that is coherent, transparent and readily interpretable. Authors should clearly identify relationships between linked publications, while reviewers and editors should assess whether secondary outputs provide distinct and meaningful contributions. Institutions and funders should support research assessment practices that recognize transparency, reproducibility, data sharing and clinical relevance rather than publication volume alone. These coordinated actions can strengthen evidence synthesis and support clinical decisions based on a more complete and reliable research record.

## Data Availability

Data sharing is not applicable to this article as no new data were created or analyzed in this study.
